# Masked polycythemia vera presenting as non-cirrhotic portal hypertension with isolated gastric varices: a case report

**DOI:** 10.1186/s12876-026-05111-x

**Published:** 2026-07-13

**Authors:** Hammam Jallad, Ayman Khaled, Heba Qabaja, Yahya Ayyash Qabaja, Mohammad Qabaja, Amjad Awartani, Ibrahim Barham, Mohammad Yaseen

**Affiliations:** 1 Department of Medicine, Faculty of Medicine and Allied Health Sciences, An-Najah National University, Nablus, Palestine; 2 Department of Medicine, Faculty of Medicine and Health Sciences, Al- Quds University, Jerusalem, Palestine

**Keywords:** Polycythemia vera, Non-cirrhotic portal hypertension, Isolated gastric varices, JAK2 V617F, Case report

## Abstract

**Background:**

Myeloproliferative neoplasms (MPN) are an uncommon but important cause of non-cirrhotic portal hypertension (NCPH) and may present with variceal bleeding rather than overt hematologic abnormalities. Diagnosis may be further delayed when polycythemia vera (PV) is masked by iron deficiency or prior venesection, resulting in non-elevated hemoglobin despite an underlying JAK2-positive neoplasm. We report a rare case of isolated gastric variceal bleeding as the presenting manifestation of presinusoidal NCPH in a patient with early myelofibrosis evolving from iron-deficient PV.

**Case presentation:**

A 57-year-old man presented with intermittent rectal bleeding and melena. His history was notable for prior erythrocytosis managed with recurrent therapeutic venipunctures and superficial thrombophlebitis. Upper endoscopy revealed isolated gastric varices without esophageal varices. Abdominal computed tomography showed massive splenomegaly with dilated portal and splenic veins, a radiologically normal liver, and no portal or splenic vein thrombosis. Laboratory evaluation showed hemoglobin of 13.2 g/dL and normal liver chemistries. The patient underwent transjugular liver biopsy with concurrent hepatic venous pressure gradient (HVPG) measurement, which demonstrated a normal gradient of 5 mmHg, while histopathology revealed focal hepatic extramedullary erythropoiesis without cirrhosis. JAK2 V617F testing was positive. Peripheral blood smear and bone marrow biopsy showed leukoerythroblastosis, absent iron stores, panmyelosis, atypical megakaryocytic clustering, and grade 1–2 reticulin fibrosis, consistent with early myelofibrosis evolving from iron-deficient polycythemia vera.

**Conclusions:**

This case highlights that masked MPN should be considered in patients with non-cirrhotic portal hypertension, massive splenomegaly, or variceal bleeding even when hemoglobin is normal at presentation. Early JAK2 testing and bone marrow evaluation are essential for diagnosis.

**Supplementary Information:**

The online version contains supplementary material available at https://doi.org/10.1186/s12876-026-05111-x.

## Introduction

MPN are an uncommon but important cause of NCPH and may present with splenomegaly, thrombosis, or variceal bleeding rather than overt hematologic abnormalities [1]. PV and myelofibrosis have both been associated with portal hypertensive complications through mechanisms including splanchnic thrombosis, splenomegaly, and extramedullary hematopoiesis [1–3]. Recognition may be further delayed when polycythemia vera is masked by iron deficiency or prior venesection, resulting in non-elevated hemoglobin or hematocrit despite an underlying JAK2-positive MPN [4–7]. We report a rare case of isolated gastric variceal bleeding as the presenting manifestation of presinusoidal NCPH in a patient with early myelofibrosis evolving from iron-deficient PV.

## Case presentation

A 57-year-old male presented with a four-month history of intermittent rectal bleeding and a recent episode of acute melena, which first commenced on the evening of September 27, 2025. Subsequent upper endoscopy, prompted by the melena, was performed on September 30, 2025, showing a 3-day interval between the symptom onset and the procedure. His past medical history included hypertension managed with candesartan and amlodipine, gout treated with colchicine, and chronic kidney disease with a baseline creatinine of 1.3 mg/dL. He had a history of transient hyperthyroidism (possibly in the context of prior thyroiditis), which had been previously treated and was clinically euthyroid off medications. Other short-term or supportive medications unrelated to the management of portal hypertension included acetylcysteine 600 mg three times daily after contrast-enhanced CT to reduce the risk of contrast-induced nephropathy in the setting of chronic kidney disease, acyclovir 400 mg daily for a new herpes zoster infection involving the right posterior thoracic dermatome, levofloxacin 750 mg daily for severe bacterial bronchitis as prescribed by the internal medicine team, and clonazepam 2 mg daily, which the patient used occasionally for a pre-existing sleep disorder/restless leg symptoms. His past medical history was significant for a previously documented erythrocytosis (peak hemoglobin 17 g/dL) managed with recurrent therapeutic venipunctures. Notably, he had a history of recurrent right lower limb superficial thrombophlebitis approximately one year prior to presentation. According to the treating vascular specialist, anticoagulation was prescribed because the patient had recurrent thrombophlebitis with high-risk features for thrombosis and was unable to tolerate nonsteroidal anti-inflammatory drugs, making anticoagulation the preferred treatment. This anticoagulation was prescribed for superficial thrombophlebitis only, with no prior documented portal, hepatic, or splenic vein thrombosis.

He reported no alcohol abuse or recent use of nonsteroidal anti-inflammatory drugs.

**Fig. 1:**
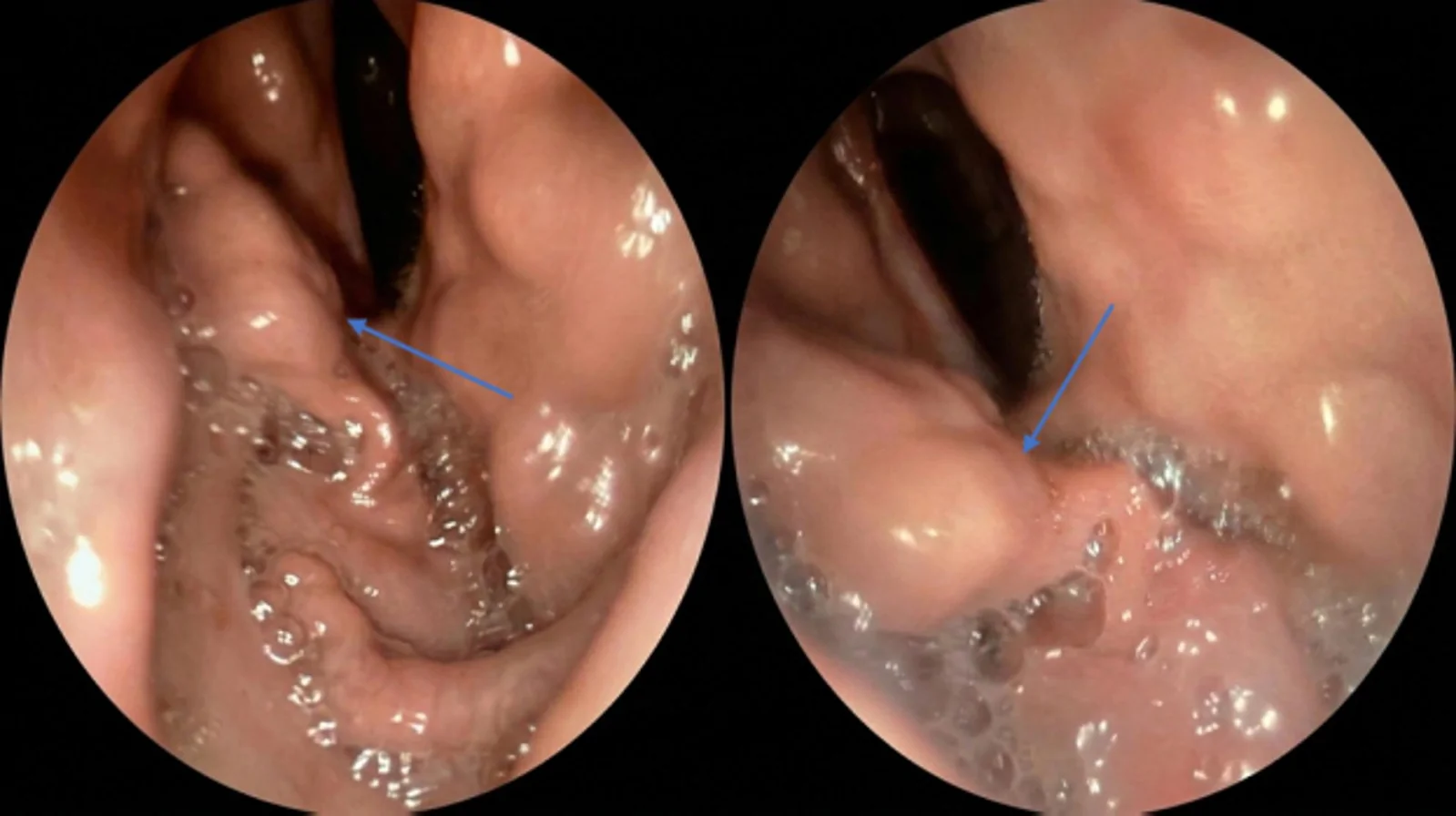
Upper endoscopy findings. Large, tortuous isolated gastric varices (IGV1) (arrows) are noted in the gastric fundus. The absence of esophageal varices and a normal hepatic venous pressure gradient (HVPG) are consistent with a pre-sinusoidal etiology of portal hypertension

A complete colonoscopy revealed sigmoid diverticulosis and a small (< 5 mm) tubular adenoma with low-grade dysplasia, which was fully removed during the procedure. Given its small size, the polyp was thought unlikely to be responsible for the patient’s bleeding. Diverticulosis was also thought to be an improbable cause of the melena, which usually manifests as hematochezia due to its distal location. Subsequent upper endoscopy, performed three days post-symptom onset (September 30, 2025), demonstrated large, tortuous isolated fundal gastric varices consistent with the Sarin classification of Isolated Gastric Varices Type 1 (IGV1) (Fig. 1), in addition to a clean-based lesser-curvature ulcer and no esophageal varices. The clean-based appearance of the ulcer made it an unlikely source of recent bleeding. According to the treating gastroenterologist, the fundal varices were the most likely bleeding source based on the endoscopic findings, particularly the presence of a clean-based ulcer with no signs of recent hemorrhage and the identification of gastric fundal varices, which were further supported by contrast-enhanced CT revealing prominent gastric wall varices. The patient’s bleeding was clinically gradual and intermittent over days to weeks, presenting as melena without hemodynamic compromise or a sudden, substantial drop in hemoglobin, which was consistent with the reported clinical course.

**Fig. 2:**
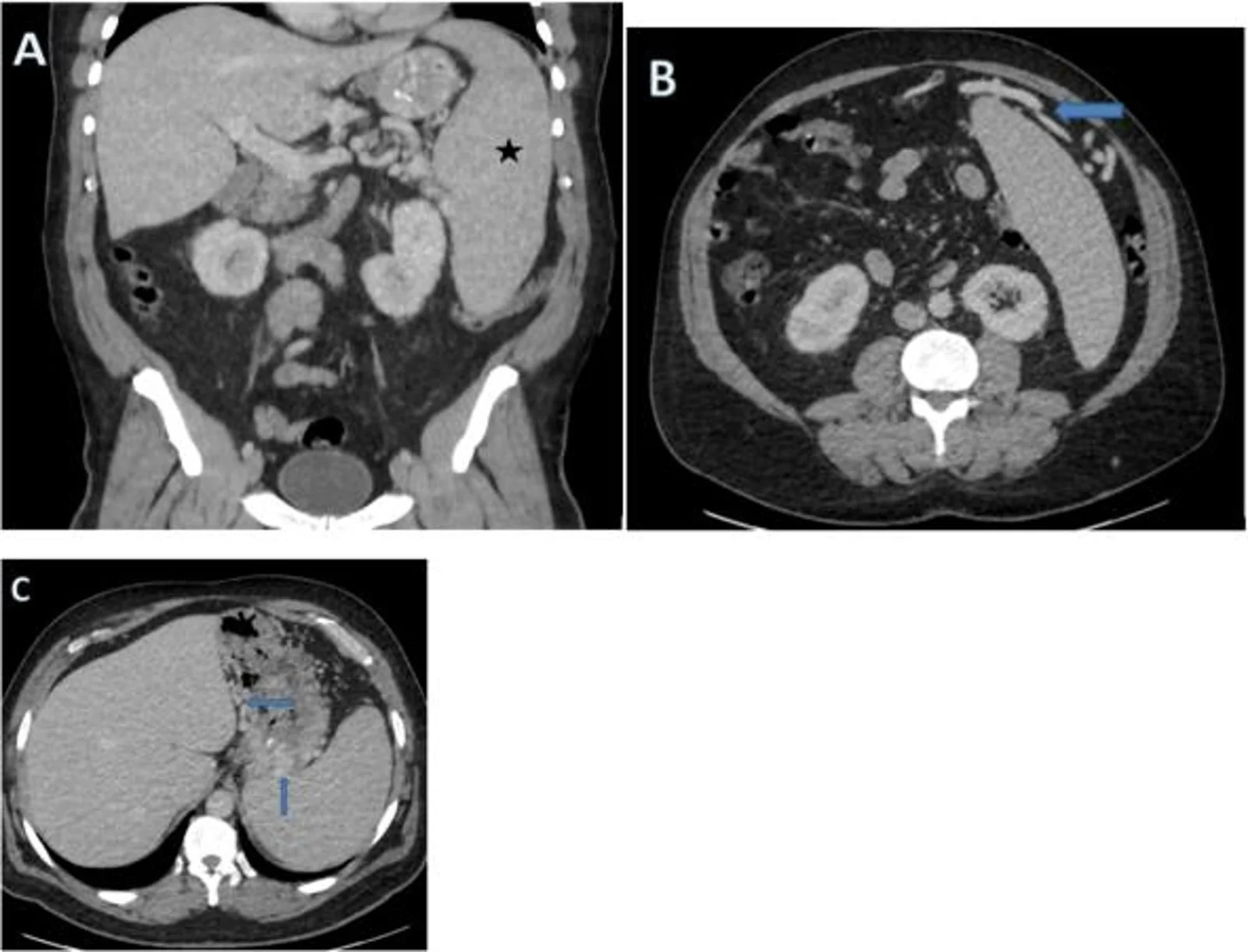
A selected coronal CT scan with intravenous contrast image showed splenomegaly (black star) (Figurer2**A**). Selected axial CT scan with intravenous contrast images showed multiple varicose veins at the perisplenic area (Fig. 2**B**) and gastric wall (Fig. 2**C**)

**Fig. 3:**
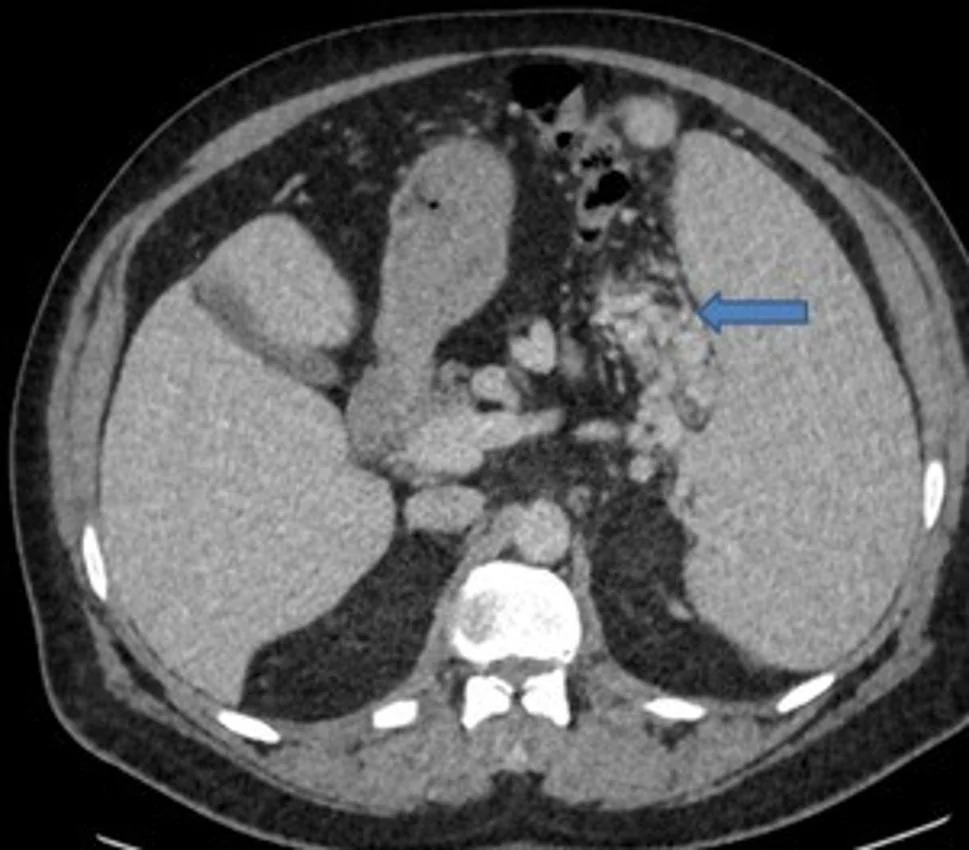
Selected axial CT scan with intravenous contrast images showed multiple varicose veins (blue arrow) at the splenic hilum

Contrast-enhanced computed tomography (CECT) of the abdomen and pelvis revealed massive splenomegaly (24 cm in bipolar dimension) with dilation of the portal, splenic, and multiple serpentine perisplenic veins. Notably, the CECT definitively demonstrated prominent, tortuous varicose veins running directly within the gastric fundal wall (Fig. 2C), providing clear radiological corroboration of the endoscopic findings. The liver appeared radiologically normal without evidence of cirrhosis, portal vein thrombosis, or splenic vein thrombosis. Additionally, the CECT clearly showed a fully patent, non-thrombosed splenic vein with complex collaterals visible at the perisplenic region (Fig. 2B) and splenic hilum (Fig. 3A). Contrast-enhanced CT demonstrated a patent splenic vein without evidence of current splenic vein thrombosis, arguing against fixed left-sided portal hypertension. Nevertheless, a transient prior splenic vein thrombotic event with spontaneous recanalization cannot be completely excluded. In addition, multidisciplinary evaluation by gastroenterology and rheumatology, including review of the major laboratory results, did not identify liver cirrhosis, hepatic or splenic venous thrombosis, systemic autoimmune/rheumatologic disease, antiphospholipid syndrome, or another secondary gastrointestinal cause to explain the portal hypertensive presentation.

Initial laboratory evaluation showed a hemoglobin level of 13.2 g/dL, a hematocrit of 42%, a white blood cell count of 7.0 × 10⁹/L, and a platelet count of 206 × 10⁹/L. Liver chemistries were normal (albumin 4.05 g/dL, total bilirubin 0.5 mg/dL, AST 19 U/L, ALT 16 U/L). There was no evidence of hemodynamic instability at presentation, supporting a chronic, slow upper gastrointestinal bleeding pattern rather than an acute massive hemorrhage. Driven by the history of thrombophlebitis and the imaging findings of non-cirrhotic portal hypertension, a hypercoagulable panel was obtained. Lupus anticoagulant was not detected, but serology was positive for anticardiolipin IgG (14.0 U/mL) and IgM. Low-titer anticardiolipin antibodies were detected but did not fulfill criteria for antiphospholipid syndrome in the absence of confirmatory persistent positivity. These findings were therefore interpreted as secondary, clinically non-definitive abnormalities and did not independently alter management. Additionally, as the patient had already started therapeutic anticoagulation with apixaban, confirmatory testing after 12 weeks to meet official APS criteria was not pursued.

Transjugular liver biopsy demonstrated an HVPG of 5 mmHg, indicating a pre-sinusoidal, non-cirrhotic etiology for portal hypertension. Transjugular liver biopsy demonstrated preserved hepatic architecture without cirrhosis. Histopathology identified focal clusters of extramedullary erythropoiesis within the hepatic parenchyma. No significant sinusoidal dilatation, sinusoidal congestion, sinusoidal fibrosis, or features of sinusoidal obstruction syndrome were identified. These findings supported a non-cirrhotic, predominantly presinusoidal mechanism of portal hypertension. Of note, transient elastography (FibroScan) was not performed as definitive histologic evaluation was successfully obtained via the liver biopsy. This finding, coupled with the history of erythrocytosis, prompted genetic testing, which confirmed a *JAK2* V617F mutation.

A thorough investigation that ruled out other reasons confirmed the diagnosis of non-cirrhotic portal hypertension. Cirrhosis was ruled out using triphasic contrast-enhanced CT, liver biopsy, normal liver biochemistry, and a specialist gastrointestinal assessment. Additional rheumatologic and cardiology examinations revealed no alternate explanation for portal hypertension.

Subsequent peripheral blood and bone marrow evaluation characterized the underlying myeloid neoplasm. The peripheral blood smear demonstrated a leukoerythroblastic picture with hypochromic, microcytic red cells, teardrop cells, nucleated red blood cells, and giant platelets. The bone marrow biopsy was markedly hypercellular (95%) with panmyelosis. Megakaryocytes were significantly increased, exhibited atypical morphology, and formed loose clusters. Marrow iron stores were absent. Reticulin staining demonstrated WHO grade 1–2/3 fibrosis without collagen deposition. Flow cytometry confirmed no excess blasts (1% myeloblasts).

The final diagnosis was early myelofibrosis, transforming from iron-deficient PV, manifesting atypically as an upper gastrointestinal bleed secondary to NCPH. Notably, this cascade occurred in the absence of classic peripheral cytopenias. The normal hemoglobin at presentation (13.2 g/dL) was attributed to the iron-deficient state induced by gastrointestinal bleeding and prior therapeutic venipunctures, which masked the underlying erythrocytosis.

For acute management, the patient underwent successful endoscopic band ligation of the fundal varices, achieving immediate hemostasis. Following the diagnosis of post-PV early myelofibrosis, the hematology service initiated targeted cytoreduction with ruxolitinib 20 mg twice daily.

During the initial diagnostic evaluation, before the underlying cause of portal hypertension was established, the gastroenterology team empirically administered propranolol 10 mg twice daily as temporary portal-hypertension-directed therapy. Spironolactone 25 mg daily was used briefly during the initial evaluation but was not continued as a specific long-term therapy for variceal prophylaxis, as the patient had no documented ascites or fluid retention. Propranolol was not titrated because it was only supposed to be used temporarily during the diagnostic workup and was withdrawn after the underlying cause of the portal hypertension was identified. The patient also continued his chronic medications for pre-existing comorbidities, including allopurinol for recurrent gout and rosuvastatin for familial dyslipidemia. Other short-term medications unrelated to the management of portal hypertension included acetylcysteine following contrast-enhanced CT to reduce the risk of contrast-induced nephropathy, acyclovir for herpes zoster infection, levofloxacin for bacterial bronchitis, and clonazepam for restless leg symptoms.

The patient maintained a constant hemoglobin level of 9.1 g/dL and was clinically stable at the 5-month follow-up, with no recurrent bleeding or thrombotic episodes. Monitoring for bone marrow suppression, tracking renal function, and routine endoscopic surveillance of the varices are all part of the continuing care plan (Table 1).

The patient was informed about the diagnostic findings, treatment plan, and follow-up strategy, and provided written informed consent for publication of this case report and accompanying images. A formal patient-authored perspective statement was not obtained.

**Table 1:** Iron profile and hematological parameters during follow-up

Variable	Value	Reference range
Serum Iron	96 µg/dL	60–170 µg/dL
Hb	9.1 g/dL	13.5–17.5 g/dL
MCV	79 fL	80–100 fL
MCH	25.7 pg	27–33 pg
Ferritin	84.93 µg/dL	30–400 ng/mL
Total Iron Binding Capacity (TIBC)	273 µg/dL	240–450 µg/dL
Transferrin Saturation (TSAT)	35.2%	20–50%

## Discussion

The present case describes a 57-year-old man whose workup for melena revealed IGV, massive splenomegaly, a radiologically non-cirrhotic liver, no portal or splenic vein thrombosis, a normal HVPG of 5 mmHg, focal hepatic extramedullary erythropoiesis, JAK2 V617F positivity, and bone marrow findings consistent with early myelofibrosis evolving from iron-deficient PV. This combination is unusual because it links non-cirrhotic presinusoidal portal hypertension with a masked erythrocytotic MPN, rather than with primary chronic liver disease or overt splanchnic venous thrombosis [1, 8].

The differential diagnosis of non-cirrhotic portal hypertension includes prehepatic causes such as portal or splenic vein thrombosis, intrahepatic causes such as porto-sinusoidal vascular disorder, infiltrative disease, nodular regenerative hyperplasia, schistosomiasis, sarcoidosis, congenital hepatic fibrosis, and sinusoidal obstruction syndrome, and posthepatic causes such as Budd-Chiari syndrome, inferior vena cava obstruction, and constrictive pericarditis. Diagnosis requires integration of liver biochemistry, contrast-enhanced imaging of the portal, splenic, and hepatic veins, endoscopic findings, HVPG measurement, and liver biopsy. In this patient, CT showed a normal liver with patent portal and splenic veins and no hepatic venous thrombosis, liver chemistries were normal, HVPG was 5 mmHg, and liver biopsy showed preserved architecture without cirrhosis. Multidisciplinary evaluation did not identify autoimmune/rheumatologic disease, antiphospholipid syndrome, chronic liver disease, or another secondary gastrointestinal cause. Therefore, NCPH was attributed most plausibly to MPN-associated presinusoidal portal hypertension.

To better explain this presentation, MPN-associated non-cirrhotic portal hypertension should be viewed as a multifactorial process rather than the result of a single obstructive lesion. Portal hypertension is an uncommon but recognized complication of Philadelphia-negative MPNs, particularly polycythemia vera and myelofibrosis, and may lead to clinically significant complications such as variceal bleeding and ascites [1]. Although macrovascular splanchnic thrombosis is a well-recognized mechanism in MPN-associated portal hypertension, the absence of current portal or splenic vein thrombosis in our patient makes a purely large-vessel thrombotic explanation less likely. Therefore, alternative MPN-related mechanisms must be considered.

One important mechanism is microvascular thrombosis or obliterative injury involving small intrahepatic portal venous branches. JAK2-positive MPNs are associated with a prothrombotic state that may promote recurrent microvascular thrombosis, loss or narrowing of small portal venous radicles, and localized parenchymal remodeling, thereby increasing presinusoidal vascular resistance even when the major portal and splenic veins remain patent [1, 9]. This mechanism is clinically relevant in the present case because it provides a plausible explanation for portal hypertensive manifestations despite the absence of radiologically visible portal or splenic vein thrombosis.

A second contributing mechanism is increased portal inflow from marked splenomegaly. The patient’s massive splenomegaly likely increased splenic venous return and contributed to a hyperdynamic portal inflow state. Although increased inflow alone may not fully explain clinically significant portal hypertension, it can amplify portal pressure when superimposed on MPN-related microvascular portal venopathy or intrahepatic presinusoidal resistance [1, 2].

A third mechanism is hepatic extramedullary hematopoiesis. In this patient, liver biopsy demonstrated focal hepatic extramedullary erythropoiesis with preserved architecture and no cirrhosis. This finding provides direct histopathologic support for hepatic involvement by the underlying myeloproliferative disorder. Extramedullary hematopoietic foci may distort small portal venous or sinusoidal channels and contribute to presinusoidal resistance, particularly in myelofibrosis-associated portal hypertension [1, 10]. Taken together, the most likely pathophysiologic model in this case is synergistic: increased portal inflow from massive splenomegaly occurring on a background of MPN-related microvascular portal venopathy and focal hepatic extramedullary hematopoiesis. This also explains the normal HVPG of 5 mmHg, because HVPG may remain normal or only mildly elevated when the principal site of increased resistance is presinusoidal rather than sinusoidal [8].

The endoscopic pattern is also important because isolated fundal gastric varices without esophageal varices classically raise suspicion for left-sided portal hypertension due to splenic vein obstruction [11]. In the present case, persistent left-sided portal hypertension due to splenic vein obstruction was considered unlikely because contrast-enhanced CT demonstrated a patent splenic vein without current splenic vein thrombosis, no portal vein thrombosis, no hepatic venous thrombosis, and a radiologically non-cirrhotic liver. This was further supported by transjugular liver biopsy showing preserved hepatic architecture without cirrhosis and by a normal HVPG, consistent with a presinusoidal rather than cirrhotic sinusoidal mechanism [8]. Multidisciplinary gastroenterology and rheumatology assessment, based on the clinical findings and major laboratory results, did not identify an alternative chronic liver disease, autoimmune/rheumatologic disorder, antiphospholipid syndrome, or secondary gastrointestinal cause. Therefore, the isolated gastric varices were interpreted as part of MPN-associated presinusoidal non-cirrhotic portal hypertension rather than fixed isolated left-sided portal hypertension. Nevertheless, a transient prior splenic vein thrombotic event with spontaneous recanalization cannot be completely excluded.

Regarding management of the gastric varices, the patient underwent endoscopic band ligation during the index therapeutic endoscopy, achieving immediate hemostasis. Although cyanoacrylate injection and EUS-guided therapy are commonly used for fundal gastric varices, band ligation was the modality performed in the acute setting and successfully controlled the bleeding. Therefore, cyanoacrylate injection, EUS-guided therapy, BRTO, and TIPS were not pursued during the acute episode. Advanced endoscopic or radiologic interventions would be considered if bleeding recurs, if endoscopic control fails, or if follow-up evaluation demonstrates persistent high-risk varices requiring escalation.

Among published case reports, the closest clinical parallel is the report by Giszas et al., who described a patient with JAK2-positive primary myelofibrosis and recurrent isolated gastric variceal bleeding without portal or splenic vein thrombosis, which closely matches the non-thrombotic presentation of our case [12]. Their case supports the concept that isolated gastric variceal bleeding can be the presenting manifestation of an underlying JAK2-positive MPN [12]. However, unlike our patient, their case represented established primary myelofibrosis, whereas our case reflects a masked polycythemia vera phenotype entering early fibrotic evolution [12]. Other published cases of MPN-associated portal hypertension have typically involved concurrent splanchnic vein thrombosis [13, 14], which was explicitly absent in our patient, supporting a non-thrombotic, presinusoidal mechanism in our case. A summary of published cases with similar presentations is provided in Table 2.

**Table 2:** Selected published reports of JAK2-positive myeloproliferative neoplasms presenting with portal hypertension and gastric or esophagogastric varices

Author & Reference	Patient Age / Sex	Initial Presentation	JAK2 V617F Status	Portal Hypertension Etiology / Key Findings	Management and outcome
Link et al. [13]	Case 1: 35/male Case 2: 35/male	Case 1: Severe abdominal pain, portal and splenic vein thrombosis, esophageal and gastric varices. Case 2: Abdominal pain, recurrent chills, splenomegaly, portal vein cavernous transformation, high-grade esophageal varices.	Positive	NCPH secondary to splanchnic/portal vein thrombosis associated with occult JAK2-positive MPN. Splenomegaly and cavernous transformation of the portal vein were present. One patient had ET; the other developed prefibrotic myelofibrosis.	Repeated endoscopic variceal ligation, anticoagulation, propranolol. The second patient additionally received ruxolitinib with reduction in variceal interventions. Both achieved stable long-term follow-up under hematologic and gastroenterologic monitoring
Giszas et al., 2022 [12]	36 / Female	First upper GI bleeding, followed by recurrent life-threatening bleeding from isolated gastric varices	Positive	Primary myelofibrosis with splenomegaly; isolated gastric varices attributed to left-sided portal hypertension	Multiple cyanoacrylate injections initially failed; bleeding controlled with JAK-inhibitor therapy plus EUS-guided coils and cyanoacrylate.
Kono et al., 2015 [14]	28 / Male	Hematemesis from esophageal variceal rupture; later found to have gastric varices in the cardia	Positive	Polycythemia vera diagnosed after portal-vein occlusion, massive splenomegaly, collateral vessels, and esophagogastric varices; CBC was relatively masked at presentation	Treated with hydroxyurea; blood counts decreased and varices remained stable without further bleeding.
Present Case	57 / Male	Intermittent rectal bleeding and melena	Positive	Masked PV with concomitant iron deficiency; non-cirrhotic portal hypertension, isolated gastric varices (IGV1), massive splenomegaly, and early myelofibrosis.	Endoscopic band ligation, ruxolitinib 20 mg twice daily, and apixaban 5 mg twice daily. Stable hemoglobin levels and no recurrence of bleeding or thrombosis at follow-up.

Another important comparison is the case reported by Pati et al., in which polycythemia vera was masked by iron deficiency in a patient presenting with portal hypertension due to extrahepatic portal venous obstruction [5]. This report is conceptually important because it demonstrated that normal hemoglobin and hematocrit at presentation do not exclude PV when iron deficiency is present, especially if the red cell mass is increased or the clinical context is otherwise suggestive [5]. This same principle applies strongly to our patient, whose history included previous hemoglobin elevation up to 17 g/dL, recurrent therapeutic venipunctures, absent marrow iron stores, and microcytic hypochromic red cells, all of which support a previously overt erythrocytotic process that later became masked [4–6].

Iron deficiency is particularly relevant in PV because it is common at presentation and is often further aggravated by therapeutic phlebotomy, which intentionally restricts erythropoiesis and may normalize the hematocrit despite persistence of the clonal disorder [6, 7]. In this context, Aladağ et al. further showed that microcytosis with non-elevated hemoglobin or hematocrit can conceal PV, especially when JAK2 V617F positivity and PV-compatible marrow findings are present [4]. Accordingly, the marrow pattern in the present case—panmyelosis, atypical clustered megakaryocytes, absent iron stores, and grade 1–2 reticulin fibrosis—fits well with the interpretation of iron-deficient PV progressing toward early post-PV myelofibrosis [4, 7].

The presence of JAK2 V617F supports this interpretation, since the mutation is found in more than 95% of patients with PV, although it is not by itself specific because it may also occur in other classical MPNs [4, 7]. Additional support for the association between PV and non-cirrhotic portal hypertension comes from the case series by Zhang et al., who described five patients with PV-associated non-cirrhotic portal hypertension, all with splenomegaly and relatively preserved liver function, while several also had portal vein thrombosis and significant varices [2]. Compared with that series, the present case is more unusual because the bleeding source was isolated gastric rather than predominantly esophageal varices, and because the hematologic phenotype at presentation was masked rather than overtly polycythemic.

The patient’s previously documented erythrocytosis had been managed with repeated therapeutic venesection, but a complete hematologic evaluation, including JAK2 testing and bone marrow assessment, had not been performed at that time. Earlier investigation may have allowed recognition of polycythemia vera before the development of portal hypertensive complications and early fibrotic evolution. This emphasizes the importance of systematic evaluation of unexplained erythrocytosis, particularly in patients with thrombotic manifestations or splenomegaly.

Taken together, the available literature suggests that the present case occupies a distinctive intersection of three recognized but rarely coexisting phenomena: MPN-associated non-cirrhotic portal hypertension, isolated gastric variceal bleeding, and iron-deficiency-masked polycythemia vera undergoing early fibrotic transformation.

There are various limitations to this report. The development of portal hypertension and the underlying MPN cannot be conclusively linked in a single retrospective case report. Furthermore, iron deficiency and previous therapeutic venesections obscured the distinctive erythrocytotic phenotype upon presentation, making the diagnosis of masked polycythemia vera very difficult. The contribution of the positive anticardiolipin antibodies to the patient’s thrombotic risk or portal hypertension symptoms is still unknown because they were of low titer and were not deemed clinically significant. Despite these limitations, diagnosis is well supported by the combination of clinical, radiologic, histopathologic, and molecular results.

The main clinical message is that an underlying MPN should be considered in patients with non-cirrhotic portal hypertension, massive splenomegaly, or variceal bleeding even when the hemoglobin is normal at presentation, particularly if there is a history of prior erythrocytosis, venesection, or thrombosis. Early JAK2 testing and bone marrow evaluation may therefore be essential for identifying a masked MPN before the development of further portal hypertensive or thrombotic complications.

## Conclusion

This case highlights an unusual presentation of JAK2 V617F–positive myeloproliferative neoplasm with isolated gastric variceal bleeding due to presinusoidal non-cirrhotic portal hypertension. It also illustrates how iron deficiency and prior therapeutic venesection may mask polycythemia vera, delaying diagnosis until complications occur. Clinicians should consider early JAK2 testing and bone marrow evaluation in patients with unexplained non-cirrhotic portal hypertension, massive splenomegaly, unexplained varices, prior erythrocytosis, or thrombotic events, even when hemoglobin and hematocrit are not elevated.

## Supplementary Information

Supplementary Material 1.

## Data Availability

All data supporting the findings of this case report are included within the article. Additional de-identified clinical information may be available from the corresponding author upon reasonable request and were permitted by institutional and patient-confidentiality requirements.

## Abbreviations

APS: Antiphospholipid syndrome
CECT: Contrast-enhanced computed tomography
Hb: Hemoglobin
HVPG: Hepatic venous pressure gradient
IGV1: Isolated Gastric Varices Type 1
JAK2: Janus kinase 2
MCV: Mean corpuscular volume
MCH: Mean corpuscular hemoglobin
MPN: Myeloproliferative neoplasms
NCPH: Non-cirrhotic portal hypertension
PV: Polycythemia vera
TIBC: Total iron binding capacity
TSAT: Transferrin saturation
WHO: World Health Organization
