# Supplementary material for: Masked polycythemia vera presenting as non-cirrhotic portal hypertension with isolated gastric varices: a case report
Source: BMC Gastroenterol. 2026 Jul 13;26:618. doi: 10.1186/s12876-026-05111-x (PMC13644044; doi:10.1186/s12876-026-05111-x)
Supplement: Supplementary file 1 [file 12876_2026_5111_MOESM1_ESM.pdf]

CARE case report guidelines

CARE Checklist of information to include when writing a case report

(cc) BY-NC-ND

Topic	Item	Checklist item description	Reported on Line
Title	1	The diagnosis or intervention of primary focus followed by the words "case report"	p1:l1-2
Key Words	2	2 to 5 key words that identify diagnoses or interventions in this case report, including "case report"	p2:l5-6
Abstract (no references)	3a	Introduction: What is unique about this case and what does it add to the scientific literature?	p1:l12-18
	3b	Main symptoms and/or important clinical findings	p1:l19-31
	3c	The main diagnoses, therapeutic interventions, and outcomes	p1:l25-31; p4:l13-25
	3d	Conclusion—What is the main "take-away" lesson(s) from this case?	p2:l1-4
Introduction	4	One or two paragraphs summarizing why this case is unique (**may include references**)	p2:l13-22
Patient Information	5a	De-identified patient specific information.	p2:l24-25
	5b	Primary concerns and symptoms of the patient.	p2:l24-25; p3:l1-9
	5c	Medical, family, and psycho-social history including relevant genetic information	p2:l27-36; p3:l35-p4:l7
	5d	Relevant past interventions with outcomes	p2:l32-35
Clinical Findings	6	Describe significant physical examination (PE) and important clinical findings.	PE NR; p3:l21-p4:l7
Timeline	7	Historical and current information from this episode of care organized as a timeline	p2:l24-p4:l28
Diagnostic Assessment	8a	Diagnostic testing (such as PE, laboratory testing, imaging, surveys).	p3:l1-p6:l5
	8b	Diagnostic challenges (such as access to testing, financial, or cultural)	p1:l14-18; p8:l12-34; p10:l9-16
	8c	Diagnosis (including other diagnoses considered)	p3:l1-20; p4:l8-12
	8d	Prognosis (such as staging in oncology) where applicable	p4:l4-12; p4:l22-25
Therapeutic Intervention	9a	Types of therapeutic intervention (such as pharmacologic, surgical, preventive, self-care)	p4:l13-21
	9b	Administration of therapeutic intervention (such as dosage, strength, duration)	p4:l13-21
	9c	Changes in therapeutic intervention (with rationale)	p4:l13-15
Follow-up and Outcomes	10a	Clinician and patient-assessed outcomes (if available)	p4:l22-28
	10b	Important follow-up diagnostic and other test results	p4:l22-p5:l3
	10c	Intervention adherence and tolerability (How was this assessed?)	NR
	10d	Adverse and unanticipated events	p4:l22-25
Discussion	11a	A scientific discussion of the strengths AND limitations associated with this case report	p10:l9-16
	11b	Discussion of the relevant medical literature **with references**.	p6:l7-p10:l8
	11c	The scientific rationale for any conclusions (including assessment of possible causes)	p7:l9-p8:l38; p10:l9-16
	11d	The primary "take-away" lessons of this case report (without references) in a one paragraph conclusion	p10:l17-30
Patient Perspective	12	The patient should share their perspective in one to two paragraphs on the treatment(s) they received	p4:l26-28
Informed Consent	13	Did the patient give informed consent? Please provide if requested	p4:l26-28; p10:l36-39 ☒ No ☐
